# Hypercalcemia due to Co-Occurring Metastatic Breast Cancer and Primary Hyperparathyroidism

**DOI:** 10.7759/cureus.16647

**Published:** 2021-07-26

**Authors:** Mohammad U Khubaib, Rania Fadlalla, Javaria Ahmad, Zainab Naseer, Hussein Mhanna

**Affiliations:** 1 Sports Medicine, Active Orthopedics & Sports Medicine, Hackensack, USA; 2 Internal Medicine, Hackensack Meridian Health Mountainside Medical Center, Montclair, USA; 3 Internal Medicine, Shalamar Medical & Dental College, Lahore, PAK; 4 Internal Medicine, Faisalabad Medical University, Faisalabad, PAK

**Keywords:** hypercalcemia, hyperparathyroidism, cancer, breast cancer, skeletal metastases, endocrinlogy, adenoma, oncology, nephrology, hormone

## Abstract

Hyperparathyroidism and malignancy are both causes of hypercalcemia. Breast cancer patients usually have hypercalcemia due to metastases or paraneoplastic syndrome resulting from ectopic PTHrP production. Occasionally, other causes such as primary hyperparathyroidism may co-exist and contribute to the hypercalcemia as well. We present the case of a 61 year old with a history of breast cancer with bony metastasis who presented with a corrected calcium level of 17.9 mg/dl. Bloodwork and imaging was suggestive of primary hyperparathyroidism. This case highlights the rare co-existence of dual etiologies of hypercalcemia and provides an overview of the presentation, diagnostic approach and management in such scenarios.

## Introduction

The normal range for serum Calcium is from 8.8-10.8 mg/dL. A level higher than that, or two standard deviations above the mean value is called hypercalcemia. Hypercalcemia is classified as mild (up to 11.9 mg/dL), moderate (up to 13.9 mg/dL) and hypercalcemic crisis (at levels greater than, or equal to 14 mg/dL). Signs and symptoms usually start to manifest with moderate hypercalcemia and most commonly include nausea, vomiting, constipation, bone pain, renal stones (leading to colic), lethargy, confusion and cardiovascular rhythm abnormalities including abnormal PR, QT, QRS, tachycardia and bradycardia [1]. Among the causes of hypercalcemia, primary hyperparathyroidism and malignancy are the most common [2]. Patients with metastatic breast cancer presenting with hypercalcemia usually have hypercalcemia secondary to the malignancy [2]. We present a rare case in which primary hyperparathyroidism and breast cancer metastatic to bones co-existed, contributing to severe hypercalcemia.

## Case presentation

**Figure 1:**
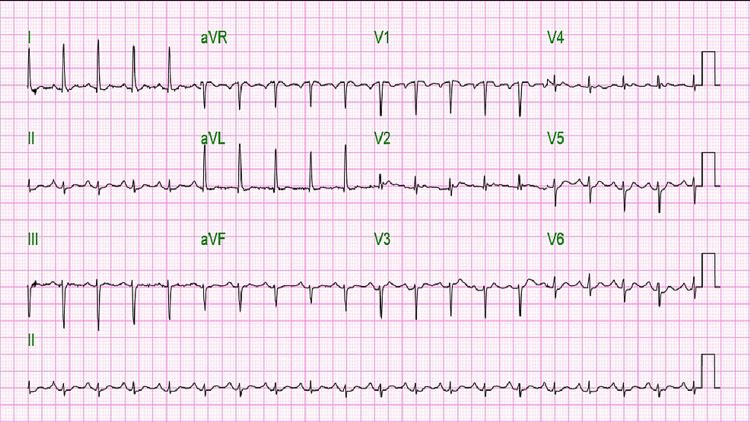
EKG of patient with hypercalcemia showing high heart rate of 108 beats per second, long PR interval of 208 milliseconds, high normal QTc interval of 392 milliseconds and short QRS complex of 78 milliseconds.

This is the case of a 61 year old Haitian female who presented to the Emergency Department with tachycardia (Figure 1), confusion, constipation and leg pain. The patient had a history of breast cancer with bony metastasis, and her bloodwork showed a corrected calcium elevated at 17.9 mg/dl. She received extensive fluid resuscitation, Zoledronate and Calcitonin, the combination of which normalized the calcium levels and improved her symptoms. On further evaluation, her PTHrP (Parathyroid Hormone Related Protein) was found to be normal and PTH (Parathyroid Hormone) came back elevated at 327 pg/ml (normal: 14 to 65 pg/mL), suggesting primary hyperparathyroidism. Furthermore, an ultrasound of the parathyroid gland showed a nodule suggestive of parathyroid adenoma. After an interdisciplinary meeting, the patient was not deemed a good surgical candidate given her extensive disease and co-morbidities, while a trial of cinacalcet also had to be discontinued due to hypocalcemia. Eventually, given her worsening metastatic breast cancer and poor prognosis, the patient decided to opt for comfort care.

## Discussion

This case highlights the possible coexistence of dual etiologies of hypercalcemia. Hypercalcemia of malignancy usually has higher serum calcium levels equal to or greater than 13 mg/dl and PTH levels are usually suppressed [3]. In parathyroid adenoma, calcium levels are usually equal to or less than 11 mg/dl and PTH levels are high [4]. Signs and symptoms of hypercalcemia are similar regardless of the etiology and include abdominal pain, bone pain, confusion, depression, weakness, kidney stones and abnormal heart rhythms including cardiac arrest [5]. Findings that favor a diagnosis of parathyroid adenoma include an asymptomatic patient with chronic hypercalcemia, postmenopausal women and patients with a normal physical examination [4,6]. On the other hand, in malignancy, there is usually a rapid increase in serum calcium and hence more dramatic symptoms [7]. Treatment of acute hypercalcemia depends on the serum calcium level rather than the actual cause. Severe hypercalcemia (i.e. levels greater than or equal to 14 mg/dl) is treated with intravenous hydration with normal saline and calcitonin. Zoledronic acid is often used for hypercalcemia secondary to malignancy [8]. Hemodialysis can be added in the case of very severe symptomatic hypercalcemia (calcium levels around 18-20 mg/dl) or if hypercalcemia is complicated by renal failure [8]. In a case like this with co-existing metastatic disease and parathyroid adenoma, treating the underlying malignancy and resection of the parathyroid are indicated to effectively treat the hypercalcemia [8,9]. Like any other malignancy, depending on the extent of the disease, palliative care has to be a consideration as well. 

## Conclusions

Patients with metastatic breast cancer usually have hypercalcemia secondary to malignancy, but occasionally can have co-existing primary hyperparathyroidism as a contributor. Acute management is the same independent of the cause and includes aggressive hydration, calcitonin, zoledronate and renal dialysis depending on the extent of hypercalcemia. Subsequent evaluation includes PTH, PTHrP and imaging. If an adenoma is found, surgical resection might be helpful.
